# The Role of Microarray in Modern Sequencing: Statistical Approach Matters in a Comparison Between Microarray and RNA-Seq

**DOI:** 10.3390/biotech14030055

**Published:** 2025-07-05

**Authors:** Isaac D. Raplee, Samiksha A. Borkar, Li Yin, Guglielmo M. Venturi, Jerry Shen, Kai-Fen Chang, Upasana Nepal, John W. Sleasman, Maureen M. Goodenow

**Affiliations:** 1 Molecular HIV and Host Interactions Section, National Institute of Allergy and Infectious Diseases, National Institutes of Health, 50 South Drive, Bethesda, MD 20894, USA; samiksha.borkar@nih.gov (S.A.B.); li.yin@nih.gov (L.Y.); jshen100@umd.edu (J.S.); kai-fen.chang@nih.gov (K.-F.C.); nepal.upasana@nih.gov (U.N.); maureen.goodenow@nih.gov (M.M.G.); 2 Division of Allergy and Immunology, Department of Pediatrics, Duke University School of Medicine, Durham, NC 27710, USA; guglielmo.venturi@duke.edu (G.M.V.); john.sleasman@duke.edu (J.W.S.)

**Keywords:** microarray, RNA-sequencing, non-parametric, HIV, transcriptomics, gene expression

## Abstract

Gene expression analysis is crucial in understanding cellular processes, development, health, and disease. With RNA-seq outpacing microarray as the chosen platform for gene expression, is there space for array data in future profiling? This study involved 35 participants from the Adolescent Medicine Trials Network for HIV/AIDS Intervention protocol. RNA was isolated from whole blood samples and analyzed using both microarray and RNA-seq technologies. Data processing included quality control, normalization, and statistical analysis using non-parametric Mann–Whitney U tests. Differential expression analysis and pathway analysis were conducted to compare the outputs of the two platforms. The study found a high correlation in gene expression profiles between microarray and RNA-seq, with a median Pearson correlation coefficient of 0.76. RNA-seq identified 2395 differentially expressed genes (DEGs), while microarray identified 427 DEGs, with 223 DEGs shared between the two platforms. Pathway analysis revealed 205 perturbed pathways by RNA-seq and 47 by microarray, with 30 pathways shared. Both microarray and RNA-seq technologies provide highly concordant results when analyzed with consistent non-parametric statistical methods. The findings emphasize that both methods are reliable for gene expression analysis and can be used complementarily to enhance the robustness of biological insights.

## 1. Introduction

Gene expression analysis plays an important role in evaluating the molecular mechanisms underlying cellular processes, development, health and disease [1,2,3]. RNA sequencing (RNA-seq) and microarray technologies are two well-established methods for quantifying gene expression profiles. For decades, throughout the late 1990s and early 2000s, microarray technology was the cornerstone of transcriptome profiling and the source for the bulk of the Gene Expression Omnibus (GEO) repository’s datasets during that time. The landscape of transcriptomics in GEO has shifted with the advent of RNA-seq technology, which, as of 2023, comprises 85% of all submissions [4].

While both technologies start with mRNA and polymerase chain reaction (PCR) amplification to produce cDNA, the two platforms differ greatly in their gene expression quantification technologies. Microarrays detect fluorescently labeled cDNA through hybridization to complementary sequences on a solid surface. The output is measured as a continuous variable, represented by fluorescence intensity. In contrast, RNA-seq leverages next-generation sequencing (NGS) of cDNA molecules, providing a digital readout of transcript abundance and sequence information.

Comparisons between RNA-seq and microarray technologies for gene expression yield both similar and different findings regarding the comparability of profiling by each method [5,6,7,8,9]. The gene expression discordance found in these studies may be attributed to inherent differences introduced by sample variability, preparation, and analytical approach. One study using technical replicates and simulated data to assess the differentially expressed gene (DEG) profiles found strong correlations between microarray and RNA-seq, with most discrepancies related to the different analytic algorithms for each platform [10]. Zhang et al. found that despite DEG discrepancies, microarray and RNA-seq had similar clinical endpoint predictions [11]. A comparison performed starting with the same samples and an appropriate non-parametric statistical approach may reduce gene expression discrepancies and enhance downstream pathway analyses.

In the last few years, increased application of artificial intelligence (AI), machine learning (ML), and similar approaches to transcriptomics research has offered powerful tools for data integration, spatial omics, and pattern recognition. The easy access to publicly available repositories of transcriptomic datasets may contribute to the development of increasingly sophisticated algorithms. Harnessing legacy microarray and RNA-seq studies, which often include data from hard to acquire or rare cohorts, may provide valuable resources for training AI models when analyzed appropriately.

In this comparison study using RNA-seq and microarray data, the same statistical approaches were applied to analyze the transcriptome profile of the same peripheral blood cell (PBC) samples. The goal of this study was to minimize DEG discrepancies and assess the relatedness of the functional analyses’ outputs between microarray and RNA-seq.

## 2. Materials and Methods

### 2.1. Clinical Profile of the Study Participants

The study participants were enrolled through the Adolescent Medicine Trials Network (ATN) for HIV/AIDS Intervention protocol 071/101, across 22 urban sites across in the United States and Puerto Rico (ClinicalTrials.gov; https://clinicaltrials.gov, Clinical Identifier No NCT00683579). The enrollment procedure and primary outcome results for this 3-year study have been reported [12,13,14,15]. A sub-study of 35 participants aged 18–25 years included 22 youth without HIV (YWOH) and 13 youth with HIV (YWH) (Table S1) selected based on the availability of whole blood samples for both microarray and RNA-seq analyses. The study participants across the groups were, on average, predominantly male (79%) and African American (70%). YWH were on combination antiretroviral therapy (ART) with suppressed viral loads (<50 HIV-1 RNA copies/mL plasma) and reported use of marijuana and tobacco, while YWOH reported no substance use.

### 2.2. RNA Isolation, Hybridization and Sequencing

Total intracellular RNA was isolated from whole blood cell samples collected in PAXgene Blood RNA tubes (Becton, Dickinson and Company, Franklin Lakes, NJ, USA) using PAXgene Blood RNA Kit (PreAnalytiX, Hombrechtikon, Switzerland) as previously described [14,15]. Globin mRNA was depleted using GLOBINclear Kit (Ambion, Waltham, MA, USA), and RNA quality was assessed by Agilent Bioanalyzer for an RNA Integrity Number (RIN) above 7. For microarray analysis, 100 ng aliquots of globin-reduced RNA was poly(A) selected, amplified and labeled using GeneChip 3′ IVT Express Kit (Affymetrix, Waltham, MA, USA) and hybridized to Gene Chip Human Genome U133 Plus 2.0 Array with 54,675 probes representing 20,174 genes (Affymetrix, Waltham, MA, USA) in the Interdisciplinary Center for Biotechnology Research (ICBR) at the University of Florida. For RNA-seq, 100 ng aliquots of globin-reduced RNA was processed with poly(A) mRNA Magnetic Isolation Module and NEBNext Ultra II RNA Library Prep Kit for Illumina (New England Biolabs, Ipswich, MA, USA). Libraries were uniquely barcoded and sequenced on the Illumina Hiseq 3000 platform (2 × 100 cycles) (Illumina, San Diego, CA, USA) at the ICBR, generating 50 million paired-end reads per sample.

### 2.3. Data Processing

**Figure 1:**
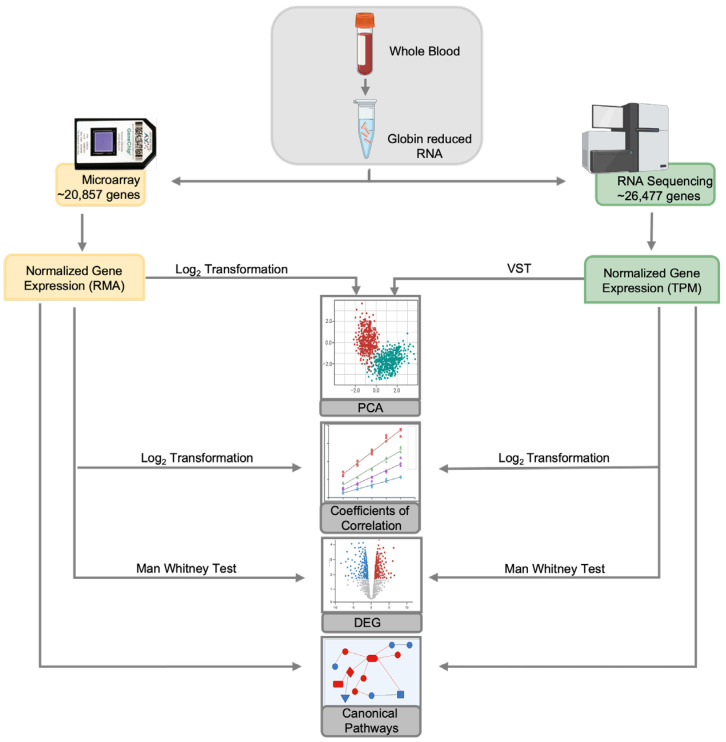
Study design. The comparison was performed by collecting aliquots from the same samples for the respective platform. Data processing included filtering low-variable probes in the microarray output and rows with no counts in the RNA-seq output. Data was normalized by log transformation for microarray and transcript per million (TPM) for RNA-seq. Principal component analysis (PCA) was performed on log-transformed data for microarray and variance-stabilizing transformation (VST) for RNA-seq. Analysis of differentially expressed genes (DEGs) was performed, and coefficient of correlation, concordance, dynamic range and unique/shared genes were derived from DEG output. Pathway analysis was performed on total gene expression data. Differential expression was determined with Mann–Whitney U (Wilcoxon rank sum) test and adjusted with Benjamini–Hochberg (BH) correction.

The study analytic workflow is outlined in Figure 1. For microarray data, the raw signal intensities in CEL format generated with Affymetrix GeneChip Operating Software were evaluated for quality by checking mean values, variance, and paired scatter plots. The raw probe signal values were background-corrected, quantile-normalized, and summarized using Robust Multi-Array Averaging (RMA) with the rma function from affy, R/Bioconductor package [16]. All expression values were converted to a log_2_ scale for downstream analysis. Microarray data were filtered by removing the lower 25% of the interquartile range (IQR) using the R package genefilter version 1.84.0 and annotated using the hgu133plus2.dg, version 3.13.0, package in R.

For RNA-seq, raw reads were checked for quality control with FASTQC [17]. Low-quality reads and residual adaptor sequences were trimmed using trimmomatic [18] and aligned to the USCS reference transcriptome, which included 26,475 annotated genes [19]. Read counts for each gene were obtained, and transcripts per million (TPM) values were calculated. The aligned reads were assessed for batch variation and outliers using BatchQC version 2.0.0 [20]. RNA-seq data were filtered by removing all genes with a sum of 0 across all samples.

### 2.4. Downstream Analysis

To assess the normality of the log-transformed microarray data, the Kolmogorov–Smirnov (KS) and the Anderson–Darling (AD) tests were employed (Table S2). To visually inspect the distributions of the microarray samples, the R package ggplot2 was used to create density plots of the data (Figure S1A). The function fitdistr from the MASS package in R was used to evaluate the goodness of fit of RNA-seq data against three distributions: normal, Poisson and negative binomial (NB) (Table S2). To visualize the distributions of the RNA-seq samples, ggplot2 was used to create overlayed density plots (Figure S1B) [21,22]. Depending on the downstream analysis, expression data was either log_2_-transformed or subjected to variance-stabilizing transformation (VST) using the DESeq2/Bioconductor R package (1.4.4.0) [23,24,25]. Log-transformed microarray and RNA-seq data were used to compute correlation coefficients using the Pearson method in R. PCA was performed on log-transformed microarray data and VST-transformed RNA-seq data using the prcomp function and visualized with the ggfortify and cluster packages in R [26,27]. Ellipses on the PCA plot represent a 95% confidence interval.

Differential expression analysis was conducted using the non-parametric Mann–Whitney U test with the wilcox.test function in R with the paired argument set to FALSE. Multiple comparisons were adjusted using the padjust function with the BH method (p_adj_ = 0.05). Dynamic fold change was calculated by dividing the means of each variable for YWOH by the means of the respective variable for YWH, followed by log_2_ transformation (Table S3). The KS test was used to determine if the distribution of the dynamic fold change was different between the two platforms (*p*-value < 0.05). Next, filtered expression values for microarray and RNA-seq data were uploaded into Qiagen’s Ingenuity Pathway Analysis (IPA) for pathway analysis [28].

## 3. Results

### 3.1. High Correlation of Gene Expression and Concordance of DEGs

After normalizing and filtering, the microarray data included 15,828 genes, about 29% less than RNA-seq’s 22,323 genes. The two platforms shared 13,577 genes, representing ~86% of microarray’s gene expression dataset and ~61% of RNA-seq’s gene expression dataset (Table 1). To further assess the concordance between RNA-seq and microarray data, differential expression analysis was performed, and outputs were compared (Table 1). RNA-seq analysis identified 2395 DEGs, while microarray analysis identified 427 DEGs. The two platforms shared 223 DEGs, representing 52.2% of total microarray DEGs and 9.3% of total DEGs by RNA-seq, with significant concordance in the overlap (*p*-value = 2.2 × 10^−16^). A Venn diagram of the DEG outputs is provided as a visualization tool (Figure S2).

**Figure 2:**
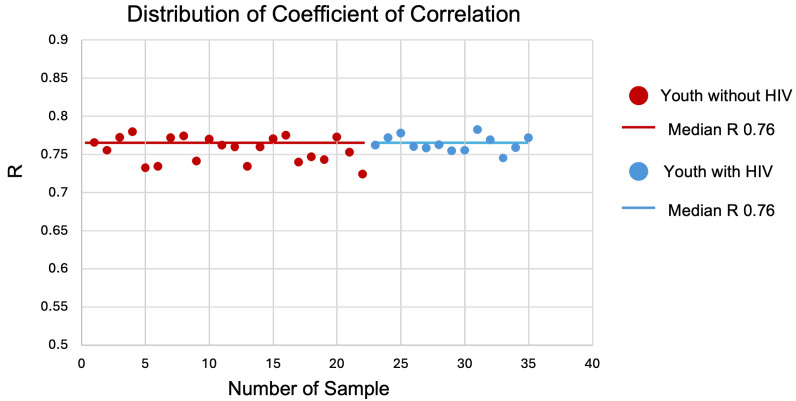
Distribution of coefficient of correlation (R). Correlation coefficient for youth with HIV (blue) and without HIV (red). The median correlation coefficient (R) is 0.76 for both groups, indicating a positive relationship between their variables.

To determine if a linear relationship between the two datasets existed, the Pearson correlation coefficient was computed on the gene expression outputs, and the distribution of the coefficient of correlation (R) was plotted for all samples (Figure 2). The median correlation coefficient (R = 0.76 and *p*-value < 0.05) signifies that the expression datasets sequenced using the two platforms show a strong correlation.

Before any downstream analyses were performed, goodness-of-fit and fit-of-distribution tests were completed (Table S2). Microarray’s distribution failed the test of normality, while the RNA-Seq data best fit the negative binomial distribution between normal, Poisson, and negative binomial.

### 3.2. PCA of Microarray and RNA-Seq

**Figure 3:**
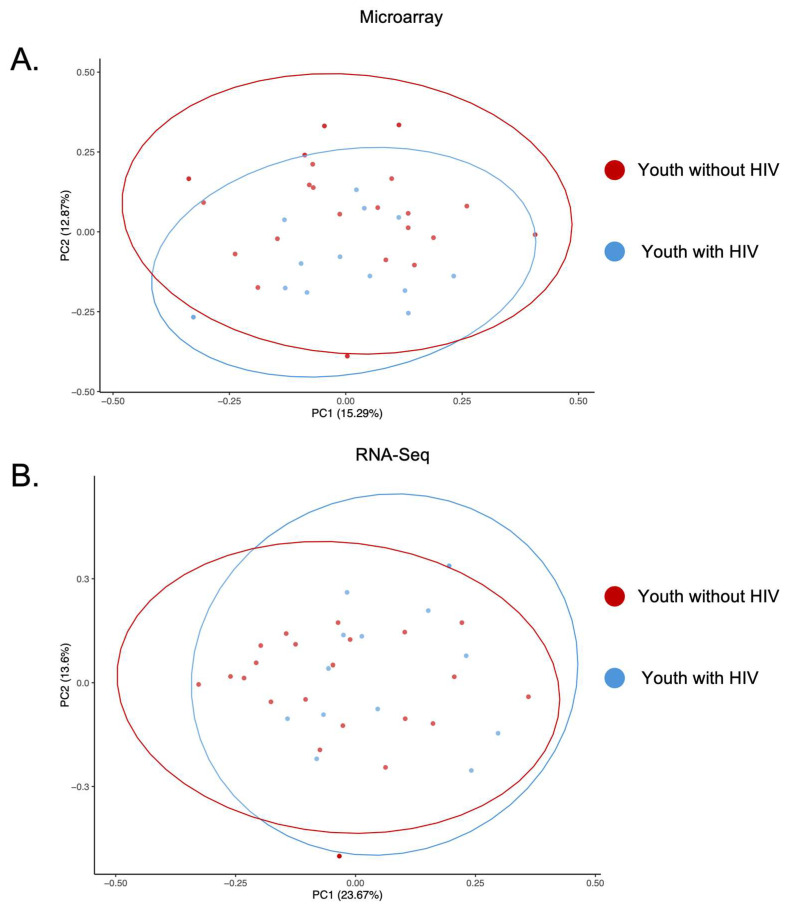
Principal component analyses. (**A**) PCA of microarray data from youth with HIV (blue) and youth without HIV (red). The first principal component (PC) accounts for 15.3% of the variability, while the second PC explains 12.9% of the variability, together representing 28.2% of the total variability in the data. (**B**) PCA of RNA-seq data from youth with HIV (blue) and youth without HIV (red). The first PC accounts for 23.7% of the variability, with the second PC explaining 13.6% of the variability, together representing 37.3% of the total variability in the data. Ellipses represent a 95% confidence interval.

To assess the variance and consistency of the two gene expression platforms, PCAs were generated using log_2_- and VST-transformed normalized expression values of the 223 shared genes (Figure 3A,B). The first two components (PC1 and PC2) accounted for 28.2% variability for PCA of microarray and 37.3% variability for PCA of RNA-seq. The PCA for each platform showed two clusters based on their biological profile. The red cluster includes all but one YWOH (22/23), while the blue cluster includes all YWH (13/13).

### 3.3. RNA-Seq Demonstrates a Greater Dynamic Range of Fold Change

**Figure 4:**
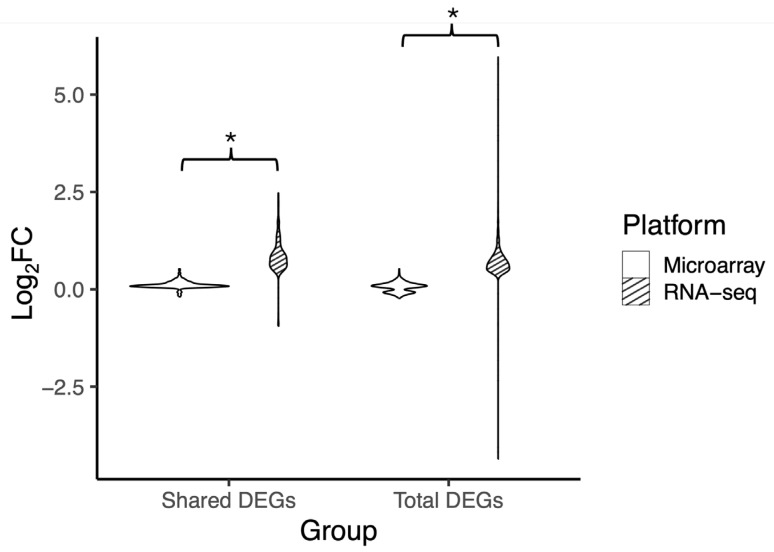
Violin plot of the range of fold change of shared and total DEGs. Log_2_FC values of DEGs common in both microarray and RNA-seq (Shared DEGs) and all DEGs in their respective platform (Total DEGs), microarray (white) or RNA-seq (striped). Log_2_FC is computed by dividing the mean of each variable in youth with HIV by the respective mean of each variable in youth without HIV and calculating the log_2_ of the output. Microarray had a range of −0.2 to 0.5 Log_2_FC and RNA-seq had a range of −0.9 to 2.5 Log_2_FC for shared DEGs. Microarray had a range of −0.2 to 0.5 Log_2_FC and RNA-seq had a range of −4.4 to 6.0 Log_2_FC for total DEGs. KS test *p*-value of less than 0.05 determined that the distributions of fold changes were significantly different, indicated by an *.

To evaluate the resolution of differential expression, comparisons of fold change dynamics between the two platforms were assessed. DEGs shared between platforms had their log_2_ fold change assessed (Table S3) and plotted (Figure 4). RNA-seq had a range between −0.9 and 2.5, while the range for DEGs from the microarray platform was between −0.2 and 0.5. The second comparison consisted of the complete set of DEGs unique to the respective platform. RNA-seq’s log_2_ fold change range was −4.4 to 6.0, while microarray had a more limited range of log_2_ fold change between −0.2 and 0.5, when compared to RNA-seq. The distribution of the fold change dynamic range was considered different by KS test *p*-value < 0.05.

### 3.4. High Concordance of Canonical Pathways

**Figure 5:**
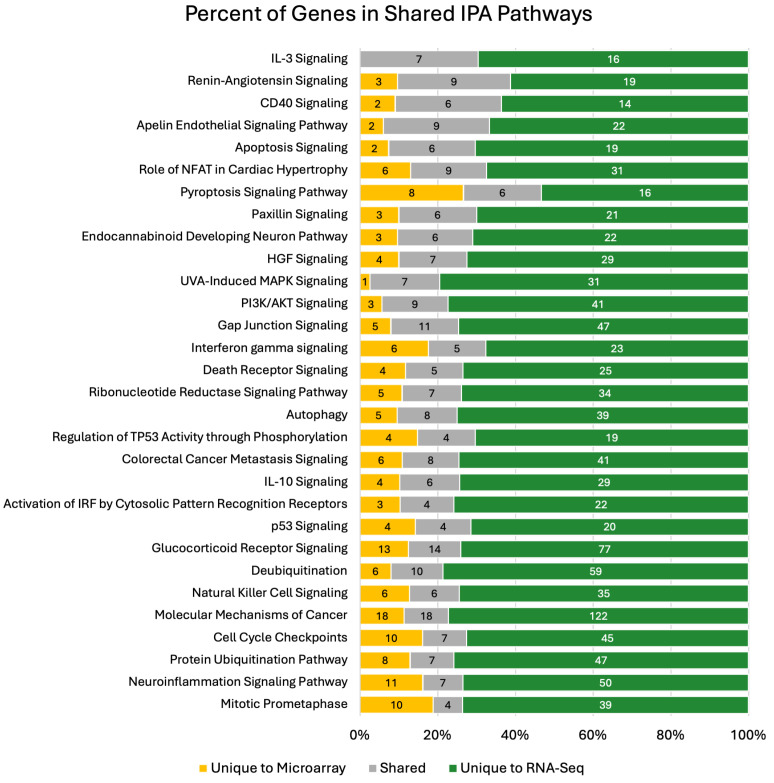
Shared pathways of IPA. IPA was used to determine functional outputs of microarray and RNA-seq data. The total number of shared pathways between microarray and RNA-seq is represented (30 pathways). The percentage of genes unique to microarray in each pathway is annotated with yellow. There is a large percentage of shared genes in each pathway, which are annotated with grey. RNA-seq had the most contributions of unique genes to shared pathways, annotated with green. The number of genes contributing to percent values is annotated in each stacked percent bar in the graph.

To determine potential biological relevance, canonical pathways were investigated using IPA on filtered expression outputs. RNA-seq analysis identified 205 perturbed pathways, while microarray analysis identified 47 pathways perturbed. Among the perturbed pathways identified by RNA-seq, 30 pathways were also detected by microarray analysis (Figure 5). The 30 shared pathways represent 14.6% of the total RNA-seq pathways and 63.8% of the microarray pathways, with significant concordance in the amount of overlap observed (*p*-value < 0.0001). The median percent of shared expressed genes across the significant pathways identified by both platforms was 15%. All significant (*p*-value < 0.001) pathways are in supplemental figures and tables (Table S4). The associated *p*-values frequently differed by orders of magnitude. For example, IL-10 signaling was enriched in both datasets, but its statistical significance was far greater in the RNA-Seq analysis (*p*-value = 2.95 × 10^−^^5^) compared to the microarray analysis (*p*-value = 7.5 × 10^−^^4^).

## 4. Discussion

In this study, we compared the results obtained from microarray and RNA-seq data, both derived from the same biological samples. The normality and goodness-of-fit analysis revealed that the microarray data had significant deviations from normality, as evidenced by the KS and AD tests. Conversely, the RNA-seq data showed a better fit with the NB distribution than the Poisson and normal distributions, reflecting the frequent issue of overdispersion in RNA-seq data where low-expressed genes’ variance exceeds the means. Using the Mann–Whitney U test for analysis ensured consistency in our statistical approach, which provided a more robust framework for comparing the outputs across different data types. The findings indicate that applying similar statistical methods to these datasets provide comparable views of canonical pathways and potential biological significance. Furthermore, the use of consistent sample preparation protocols for both RNA-seq and microarray experiments is crucial to minimize biological variability and enable a more accurate assessment of platform performance. This approach reduces variance in datasets developed from different sources, allowing for meaningful comparisons between well-curated microarray data and RNA-seq data. By using the same non-parametric statistics, we ensure a more consistent comparison between the two technologies, reducing the technical variability associated with different statistical analyses. This facilitates a fair and comprehensive evaluation of the strengths and limitations of each platform and their feasibility.

Few studies have systematically compared RNA-seq and microarray data generated from identical samples by the same statistical approach, highlighting a significant gap in the current research literature. Addressing this gap is essential to provide robust benchmarks and guide best practices in transcriptomic research. Comparative analyses hold promise to advance understanding of the factors influencing gene expression measurements. They can also improve the reproducibility and reliability of transcriptomic studies across different experimental settings and biological systems.

The Mann–Whitney U test was applied in this study due to its robustness in handling a range of varying distributions and not assuming a normal distribution [29,30], making it suitable for skewed data often encountered in RNA-seq and microarray analysis. A limitation of the method is the lack of power that parametric tests provide when the data fit their distribution [29]. Despite this limitation, we provide striking results of overlap in the reported canonical pathways.

The key findings from this study are the strong correlation of gene expression profiles determined by the coefficient of correlation, the high concordance across DEGs and canonical pathways, and the relevance and consistency of canonical pathways previously identified between the groups studied. The median correlation coefficient of 0.76 indicates a strong concordance in the gene expression profiles obtained from the two platforms. Furthermore, the comparison of DEGs between the two platforms revealed a high degree of overlap, with Fisher’s exact test confirming significant concordance. Removing the low-variance genes from the microarray dataset increased the power to detect DEGs, supporting the idea that microarray analysis benefits from focused filtering to enhance the sensitivity of differential expression analysis [31,32,33].

Two separate fold change analyses were performed: one focusing on the shared genes between the two platforms, and another using all DEGs identified for each platform individually. Restricting the analysis to shared genes limits the ability to detect platform-specific ranges, especially in RNA-seq, where more low-expressed genes are detected. However, performing fold change analysis using all DEGs may skew results, particularly in cases where one platform detects larger numbers of DEGs. The difference in dynamic fold change between the two platforms may be due to the platform-specific approaches to gene expression detection. Microarray relies on intensity probes that can be saturated with highly expressed genes, limiting low-expressed gene detection and the dynamic range of fold change. RNA-Seq can scale linearly with transcript abundance until the sequencing depth is exhausted. The results of the PCA analyses may have showcased the differences in platform-specific variability. The RNA-seq PCA analysis had a larger PC1, possibly highlighting RNA-seq’s broad range in total variability. This may be due to RNA-seq’s much broader dynamic range, which we highlight in the fold change analysis. The difference in PC1’s contribution may also be due to RNA-seq’s greater representation of total biological diversity in gene expression since microarray is optimized for predefined target sequences on the probe set.

Nested from a previous microarray study [14,15] that focused on the inflammatory impact of recreational marijuana and tobacco use, many canonical pathways perturbed in the current sub-study, based on their group function or biological role, were enriched. Pathways previously reported and identified in this study include interferon signaling, PI3K signaling, and cell death pathways. The similarity in pathway profiles between the two studies suggests that despite differences in technology, both platforms capture genes from similar pathways, and the approach could be applied to other conditions.

RNA-seq is currently the standard for gene expression data curation, leaving to question microarray’s past, present and future viability in the research community. Microarray was the largest contributor to gene expression data for many years, filling large depositories with publicly available datasets, some of which are from challenging-to-produce studies, like those involving neonates, neurological conditions, or tissue with limited availability. Machine learning (ML) algorithms and artificial intelligence (AI) tools could be created to integrate RNA-seq and microarray data, learning to recognize platform-specific biases, adjust analysis to increase confidence, and improve the predictive accuracy of biological outcomes. The high concordance of biologically relevant functional pathways and DEGs between the two platforms suggests that both technologies could serve as input for predictive models.

## 5. Conclusions

This study demonstrates that both microarray and RNA-seq technologies provide highly concordant results when analyzing the same biological samples and when analyzed with the same non-parametric statistics, such as the Mann–Whitney U test. The decreased biological variance helped to establish the robustness of the statistical analysis when comparing two different gene expression platforms. Both platforms identify similar canonical pathways and DEGs. The two platforms remain complementary tools in gene expression analysis. The results we present emphasize the importance of considering both technologies in future research, particularly in the context of AI and machine learning techniques to improve the integration of transcriptomic data for clinical and translational research. Microarray data remains a valid and relevant tool for gene expression analysis, even in an era of RNA-seq dominance.

## Figures and Tables

**Table 1:** Comparison of expressed genes and DEG numbers between microarray and RNA-seq.

	Microarray ^1^		RNA-Seq ^2^
Expressed Genes			
Unique	2251		8656
Shared	(86%)	13,577	(61%)
Total	15,828		22,323
DEGs (FDR = 0.05)			
Unique	204		2172
Shared	(52%)	223	(9%)
Total	427		2395

^1^ Microarray data were filtered by removing the genes responsible for the bottom 25% variance. ^2^ RNA-seq data were filtered by removing all genes that had sum zero across all samples.

## Data Availability

The data were uploaded to dbGaP [34] and will be released upon the publication of this manuscript (Accession ID phs002090.v1.p1, Accession ID phs002218.v1.p1).

## Supplementary Materials

The following supporting information can be downloaded at https://www.mdpi.com/article/10.3390/biotech14030055/s1: Figure S1: Density plots; Figure S2: Venn Diagram comparing DEGs identified by RNA-Seq (Blue) and microarray (Red) analysis. The Venn diagram illustrates the overlap and unique sets of DEGs detected by RNA-Seq and microarray platforms. Table S1: Demographic and clinical characteristics of study groups; Table S2: Assessment for goodness of fit for microarray’s normality and fitting of distributions for RNA-seq. Table S3. List of the shared DEGs and their respective Log_2_ fold change. Table S4A. List of the shared Canonical Pathways and their respective *p*-values. Shared pathways are highlighted in red cells. Sorted by Canonical Pathway alphabetical order. Table S4B. List of the microarray Canonical Pathways and their respective p-values. Sorted by *p*-value. Table S4C. List of RNA-Seq Canonical Pathways and their respective p-value. Sorted by *p*-value.

## Abbreviations

The following abbreviations are used in this manuscript:

RNA	Ribonucleic acid
HIV	Human immunodeficiency virus
AIDS	Acquired immunodeficiency syndrome
DEG	Differentially expressed gene(s)
GEO	Gene expression omnibus
PCR	Polymerase chain reaction
DNA	Deoxyribonucleic acid
NGS	Next-generation sequencing
PBCs	Peripheral blood cells
ATN	Adolescent Medicine Trial Network
YWOH	Youth without HIV
YWH	Youth with HIV
ART	Antiretroviral therapy
RMA	Robust multi-array averaging
IQR	Interquartile range
PCA	Principal component analysis
TPM	Transcripts per million
VST	Variance-stabilizing transformation
NB	Negative binomial
IPA	Ingenuity Pathway Analysis
KS	Kolmogorov–Smirnov
AD	Anderson–Darling
ML	Machine learning
AI	Artificial intelligence
