# Supplementary material for: The Role of Microarray in Modern Sequencing: Statistical Approach Matters in a Comparison Between Microarray and RNA-Seq
Source: BioTech (Basel). 2025 Jul 5;14(3):55. doi: 10.3390/biotech14030055 (PMC12285979; doi:10.3390/biotech14030055)
Supplement: Supplementary file 1 [file biotech-14-00055-s001.zip › biotech-3649844-supplementary.pdf]

Table S1. Demographic and clinical characteristics of study groups (n=35)

Characteristics	Youth without HIV (n = 22)	Youth with HIV (VL ≤ 50) (n = 13)
Substance use	No	M + T
Age (years)[a]	22 [20, 23]	24 [22, 25]
Male (%)	64	94
African American (%)	80	60
Days on ART	NA	940 [926, 992]
CD4 T-cells (number/μL)	755 [510, 869]	672 [432, 843]

[a] Median [25th and 75th quartile range]. Youth without HIV group was younger than youth with HIV (p=0.002). M = Marijuana, T = Tobacco.

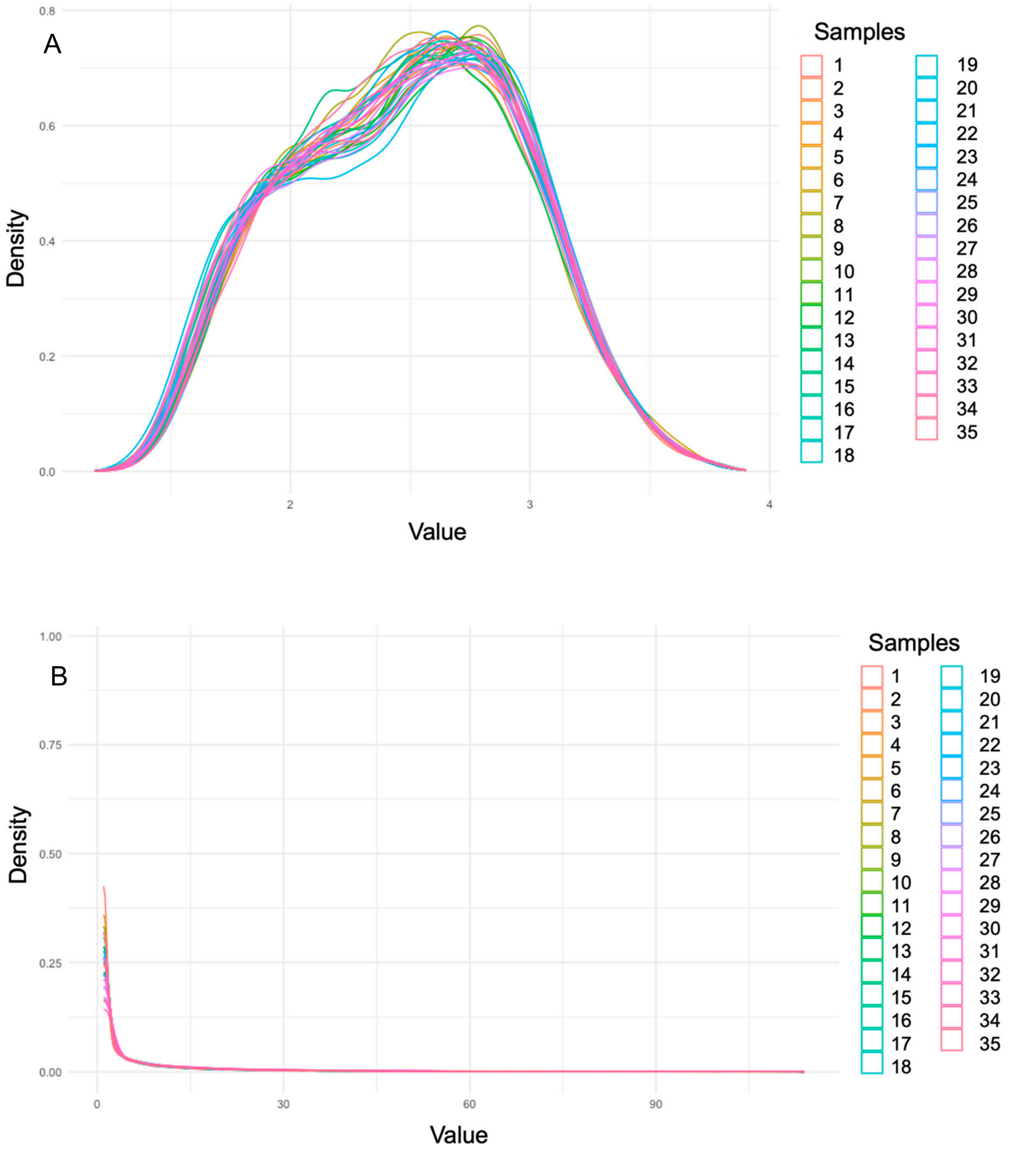

Figure S1. Density plots. A. Visual inspection of density plots of microarray using ggplot2. Visual assessment reveals a lack of normal distribution. B. Visual inspection of density plots of RNA-seq samples using ggplot2. Visual assessment reveals RNA-seq data does not deviate from a negative binomial distribution.

Table S2. Assessment for goodness of fit for microarray's normality and fitting of distributions for RNA-seq. NB negative binomial, Pois Poisson, KS Kolmogorov-Smirnov, AD Anderson-Darling.

	RNA-Seq Fit of Distributions				Microarray Goodness of Fit Statistics for Normality			
**Sample**	**Normal**	**NB**	**Pois**		**KS**	**KS pvlaue**	**AD**	**AD pvalue**
**1**	**385608**	**2027**	**40678**		**0.03**	**3.8×10^{-13}**	**31.8**	**2.2×10^{-16}**
**2**	**332593**	**2524**	**66019**		**0.03**	**2.2×10^{-16}**	**42.5**	**2.2×10^{-16}**
**3**	**388337**	**2429**	**101250**		**0.04**	**2.2×10^{-16}**	**53.0**	**2.2×10^{-16}**
**4**	**334323**	**2671**	**241297**		**0.04**	**2.2×10^{-16}**	**67.7**	**2.2×10^{-16}**
**5**	**364567**	**1961**	**72417**		**0.03**	**2.2×10^{-16}**	**41.2**	**2.2×10^{-16}**
**6**	**336087**	**3034**	**128002**		**0.04**	**2.2×10^{-16}**	**68.2**	**2.2×10^{-16}**
**7**	**333601**	**2364**	**126780**		**0.04**	**2.2×10^{-16}**	**60.7**	**2.2×10^{-16}**
**8**	**323604**	**2476**	**152200**		**0.05**	**2.2×10^{-16}**	**91.9**	**2.2×10^{-16}**
**9**	**401444**	**2044**	**24992**		**0.03**	**5.6×10^{-16}**	**45.0**	**2.2×10^{-16}**
**10**	**376926**	**2332**	**62985**		**0.03**	**4.4×10^{-15}**	**37.8**	**2.2×10^{-16}**
**11**	**346633**	**2409**	**85057**		**0.04**	**2.2×10^{-16}**	**62.6**	**2.2×10^{-16}**
**12**	**375246**	**2388**	**110528**		**0.03**	**1.2×10^{-15}**	**45.1**	**2.2×10^{-16}**
**13**	**347974**	**2346**	**40241**		**0.04**	**2.2×10^{-16}**	**61.2**	**2.2×10^{-16}**
**14**	**335240**	**2379**	**74024**		**0.04**	**2.2×10^{-16}**	**70.6**	**2.2×10^{-16}**
**15**	**370117**	**2146**	**114280**		**0.04**	**2.2×10^{-16}**	**54.0**	**2.2×10^{-16}**
**16**	**350006**	**2257**	**59567**		**0.04**	**2.2×10^{-16}**	**63.5**	**2.2×10^{-16}**
**17**	**340547**	**2308**	**123605**		**0.03**	**2.2×10^{-16}**	**48.2**	**2.2×10^{-16}**
**18**	**342737**	**2656**	**41031**		**0.03**	**3.6×10^{-15}**	**42.8**	**2.2×10^{-16}**
**19**	**339615**	**2177**	**29723**		**0.03**	**3.9×10^{-15}**	**43.5**	**2.2×10^{-16}**
**20**	**368416**	**2415**	**50319**		**0.04**	**2.2×10^{-16}**	**50.6**	**2.2×10^{-16}**
**21**	**337198**	**2361**	**64327**		**0.03**	**1.8×10^{-13}**	**30.4**	**2.2×10^{-16}**
**22**	**340437**	**2219**	**42492**		**0.04**	**2.2×10^{-16}**	**63.6**	**2.2×10^{-16}**
**23**	**341101**	**2429**	**31382**		**0.03**	**1.2×10^{-14}**	**34.4**	**2.2×10^{-16}**
**24**	**393121**	**2120**	**90296**		**0.03**	**2.2×10^{-16}**	**48.5**	**2.2×10^{-16}**
**25**	**330782**	**2549**	**194628**		**0.03**	**2.5×10^{-13}**	**28.1**	**2.2×10^{-16}**

26	333044	2562	237452	[redacted]	0.04	2.2×10^{-16}	58.2	2.2×10^{-16}
27	340707	2763	96861	[redacted]	0.04	2.2×10^{-16}	53.5	2.2×10^{-16}
28	328909	2545	82196	[redacted]	0.04	2.2×10^{-16}	64.4	2.2×10^{-16}
29	325668	2586	111230	[redacted]	0.04	2.2×10^{-16}	69.5	2.2×10^{-16}
30	331684	2562	48128	[redacted]	0.03	1.1×10^{-15}	38.1	2.2×10^{-16}
31	335619	2494	47698	[redacted]	0.04	2.2×10^{-16}	53.1	2.2×10^{-16}
32	326249	2570	56547	[redacted]	0.04	2.2×10^{-16}	60.3	2.2×10^{-16}
33	336543	2295	44698	[redacted]	0.04	2.2×10^{-16}	42.2	2.2×10^{-16}
34	332012	2493	216995	[redacted]	0.04	2.2×10^{-16}	47.7	2.2×10^{-16}
35	396203	2490	74759	[redacted]	0.03	1.1×10^{-14}	39.8	2.2×10^{-16}

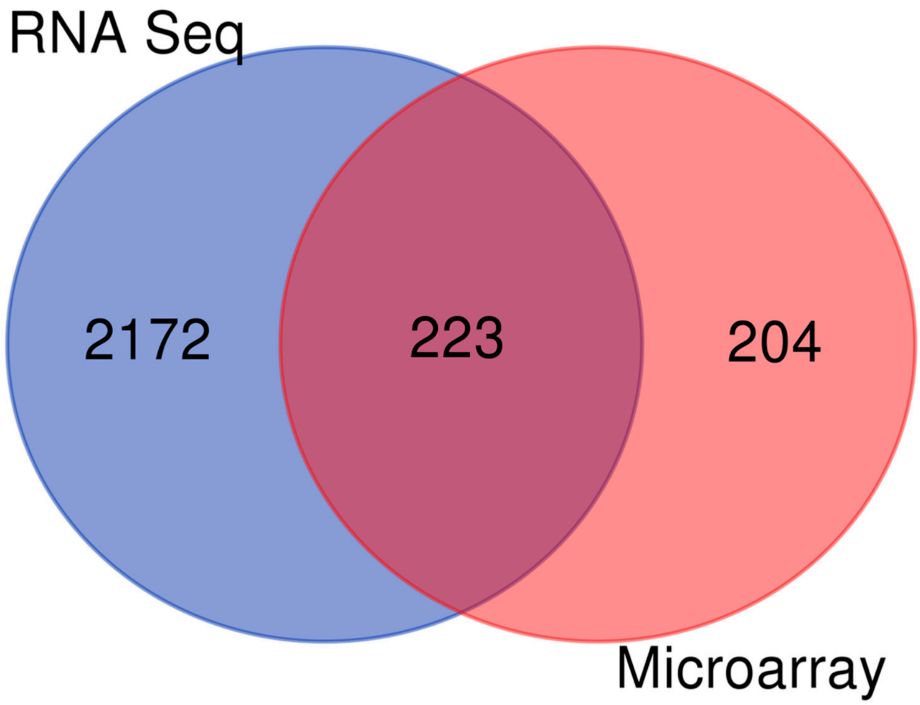

Figure S2: Venn Diagram comparing DEGs identified by RNA-Seq (Blue) and microarray (Red) analysis. The Venn diagram illustrates the overlap and unique sets of DEGs detected by RNA-Seq and microarray platforms.

Table S3. List of the shared DEGs and their respective Log_2 fold change.

**Gene**	**RNASeq log2FC**	**Microarray log2FC**
ACLY	0.65485458	0.04891292
ADCY7	0.86787566	0.04365052
ADPGK	0.47260496	0.10861006
AKAP9	0.49928633	-0.1467225
ANKIB1	0.89750529	0.09712951
ARF6	0.52698697	0.06223608
ARID5B	0.83144627	0.09712271
ARRDC4	1.72995866	0.26974122
ATE1	0.77916883	0.08979523
B3GNT2	0.85538287	0.13895933
BMF	0.61687402	0.07720893
BTG3	0.80196838	0.16922558
C1QC	2.0358187	0.22455021
C21orf91	0.84200686	0.07502941
C6orf120	0.75345501	0.09528137
CANX	0.50223191	0.05108629
CCNL1	0.61582482	0.08490374
CCNT2	0.92296322	0.12076558
CDC42SE2	0.56290701	0.05809239
CDCA7	1.75238078	0.25227064
CDK1	1.43837	0.24704762
CDK17	0.99712866	0.10471745
CDV3	0.54160102	0.03929824
CDYL2	1.12538388	0.09189855
CENPC	0.82233737	0.12870934
CEP152	0.78896513	0.10082355
CEP55	1.50451951	0.26847549
CGGBP1	0.67691877	0.08114942
CLINT1	0.76057654	0.07189472
CPEB3	0.9401072	0.12989419
CREM	0.49266891	0.08122321
CSGALNACT2	0.8549975	0.07826349
CTTNBP2NL	0.65315615	0.12948191
CUL1	0.60519159	0.06522663
CXCL10	2.21283261	0.41304346
DDX21	0.52192307	0.05427344

DDX60	1.50254557	0.19587111
DEK	0.7378386	0.08386455
DHX33	0.64780385	0.05759353
DPP8	0.60386632	0.09024052
DPYD	0.6859493	0.0792391
DUSP2	1.0100593	0.13320781
E2F7	1.71153055	0.11794234
EFR3A	0.95479315	0.07235451
EIF4G2	0.48059225	0.02714537
ENPP4	1.51088201	0.33831518
EPB41L3	1.14659965	0.18822012
EPS15	0.70676527	0.05572958
EPSTI1	1.71579569	0.2403315
EXOC1	0.6002211	0.06942617
F2RL1	0.67264133	0.15472146
FAM76B	0.79571904	0.12059729
FAS	0.97155443	0.1840186
FBXO11	0.74564535	-0.0809946
FBXO5	0.84931284	0.1772045
FBXO6	0.91150843	0.17786031
FCER2	-0.8693841	-0.1352228
FIGNL1	1.18934246	0.22576314
FLI1	0.59097404	0.08420586
FMR1	0.90477305	0.08105498
FNDC3A	0.81813189	0.05758653
FPR3	1.47901803	0.28133834
FUT4	0.57426294	0.08587247
G3BP2	0.63496306	0.06574346
GABPA	0.89739961	0.13089398
GALC	0.63989647	0.07532466
GBP3	1.34538835	0.17209881
GBP5	1.95866354	0.23866528
GCH1	1.0904779	0.10585466
GPBP1	0.60592632	0.06169657
GPD2	0.69827215	0.1168538
GPR137B	0.94598015	0.10929393
GPR15	2.47411347	0.18450433
GTF2E1	0.60185338	0.09088019
HEATR5A	0.59917765	0.12031113

HFE	0.64692872	0.05809863
HIF1A	0.75606124	0.06259627
HMGCS1	0.69027799	0.11884127
HNRNPLL	0.57758423	0.10914564
HNRNPR	0.59775231	0.05568512
ICE1	0.72264006	0.06783664
IDE	0.51891875	0.08325295
IFIH1	1.33450656	0.20288277
IL10RA	0.67258252	0.03825276
IL12RB1	0.66345644	0.06334663
IL15	0.66167917	0.21131016
IL17RC	0.57866251	0.14422817
IMPA1	0.74343608	0.11167792
ITGA4	1.3584387	0.15019162
ITSN2	0.52083216	0.05634054
KIAA1109	1.06446753	0.07402754
KLF10	1.75145675	0.22462396
KLF11	1.14698799	0.15375895
KLHL29	0.87919153	0.13371607
LACC1	0.99137829	0.18936249
LACTB	0.53691177	0.11631139
LAG3	1.29904152	0.27943682
LAMP3	1.38553935	0.41580511
LIG4	0.98241284	0.21017928
LMBRD2	1.3632331	0.10992412
MACF1	0.90948873	-0.0821683
MAP2K1	0.53159985	0.05065214
MAPK9	0.58566863	0.06958144
MBD2	0.46583647	0.04176738
MCM4	0.85189905	0.11346115
MCOLN2	1.14221437	0.15657894
MELK	1.20656424	0.20514019
MEX3C	0.91128008	0.10516747
MFAP3	0.6258325	0.07857976
MIA3	0.41416042	0.07564092
MSH6	0.47192033	0.10612822
MTHFD2	0.88495831	0.15034112
MTMR1	0.60738358	0.04063276
N4BP2L1	0.58361069	0.06034747

NAB1	0.90129387	0.10050972
NAPB	0.73485001	0.07200481
NRAS	0.78795845	0.09610639
NUAK1	2.19789146	0.27231196
NUP93	0.50282232	0.06370728
P2RX7	0.86487424	0.12335673
PANK3	0.80500607	0.0744865
PANX1	0.88040324	0.11482029
PAPSS1	0.55980929	0.0719789
PARP12	0.85179659	0.09096665
PARP14	1.28281313	0.15799727
PDE4A	0.69159051	0.06207203
PDIA3	0.41870818	0.06784276
PHTF1	0.51637542	0.12397327
PI4K2B	1.03576781	0.16909692
PIK3AP1	1.0812277	0.15293205
POLG	0.48409997	-0.0739082
PPP4R3B	0.62306604	0.10076787
PRC1	0.91142687	0.23390733
PRDM1	0.79931658	0.06612571
PRDM10	0.826044	0.06429762
PRDX3	0.44916002	0.078419
PRKAG2	0.58430876	0.07163393
PRKAR2A	0.80677366	-0.0357205
PRKCH	0.6695924	0.06535389
PRNP	0.56331135	0.09067352
PROSER1	0.69606791	0.08095444
PRR5L	0.93234674	0.1046748
PSTPIP2	0.72112018	0.12724729
PTPN12	0.59863377	0.09065539
PUM1	0.74016193	0.05271611
RAB22A	0.49161801	0.08538028
RAP2B	0.59087586	0.06268735
RCAN1	0.63805257	0.08161503
RCAN2	2.37175175	0.33175273
RCC2	0.49952859	0.04672051
RDX	0.72371875	0.10018645
RGL1	0.83353268	0.08819311
RHOU	0.86666054	0.110005

RNF146	0.53088214	0.09446659
RREB1	0.77419961	-0.0318949
RTP4	1.25250905	0.20752854
SAP30	0.49770863	0.09142518
SASH1	1.30113856	0.25084127
SEPSECS	0.80757377	0.16207725
SGPP1	1.07436153	0.19181109
SH2D1A	0.94309693	0.10286957
SIGLEC1	1.72060942	0.26870956
SLC25A20	0.59932194	0.0827565
SLC30A1	1.08590687	0.10594885
SLMAP	0.6961486	0.13261154
SMC4	0.7685721	0.10946581
SPDL1	0.76603704	0.21801601
SRBD1	0.78887843	0.10184618
SS18	0.46621776	0.12263409
SSPN	0.88715438	0.16387246
STAMBPL1	0.74882203	0.09842071
STAT1	1.46574713	0.16859134
STYK1	1.37555436	0.36469971
SUSD6	0.58469541	0.05911774
SYNCRIP	0.60596189	0.07322656
SYNJ1	0.81927234	0.06874052
TAF1	0.92812625	0.07104176
TAP2	0.88072576	0.10427086
TBK1	0.88811689	0.12621854
TCL1A	-0.8727695	-0.1884246
TDRD7	0.8115015	0.11790614
TFEC	1.28804031	0.29716346
TIGIT	1.3470841	0.18817194
TMEM165	1.19897836	0.11719498
TMEM181	0.82037055	0.08206814
TMEM87A	0.46635631	0.04880661
TMIGD2	-0.6446472	-0.1180907
TNFAIP3	1.00912249	0.07572092
TOP2A	1.17862251	0.30453307
TOPBP1	0.61814918	0.06534535
TOR1B	0.70385416	0.10642639
TP53BP2	0.66022502	0.05893395

TRAF3	0.75010522	0.04923008
TRIM22	1.226673	0.11141897
TRIM59	0.63338617	0.17893355
TRRAP	0.7350184	0.08335892
TTF2	0.97068443	0.18835317
TTTY15	1.19909132	0.19954905
TXNIP	0.88013455	0.05508403
UBR1	0.93572871	0.10125465
UBR5	0.7614357	0.08800903
UBR7	0.5698173	0.06374932
USP15	0.58840098	0.06441156
USP18	1.38015774	0.23399639
USP25	0.81687422	0.09617733
USP7	0.40634758	-0.0650911
USP9Y	1.72629789	0.52207534
UTRN	1.02351702	0.07705672
UVRAG	0.51575994	0.07906814
VCPIP1	1.0038983	0.10097545
VPREB3	-0.9478731	-0.1673795
VPS54	0.84192775	0.09528976
WDR37	0.54296497	0.03934843
WDR45B	0.48680165	0.04416706
WDR7	0.82452886	0.08233407
WEE1	0.98497303	0.15789262
XRN1	0.98822283	0.10166797
ZBTB14	0.67898057	0.06418715
ZFY	1.56758042	0.48381264
ZMYND11	0.94365757	0.08464444
ZNF684	0.82494601	0.17287411
ZNRF2	0.7620289	0.13508149
ZSCAN25	0.58943352	0.07465607

Table S4A. List of the shared Canonical Pathways and their respective p-values. Shared pathways are highlighted in red cells. Sorted by Canonical Pathway alphabetical order.

RNA		Microarray	
**Ingenuity Canonical Pathways**	**p-value**	**Ingenuity Canonical Pathways**	**p-value**
Activation of IRF by Cytosolic Pattern Recognition Receptors	1.02329×10^{-06}	Activation of IRF by Cytosolic Pattern Recognition Receptors	0.00023988
Apelin Endothelial Signaling Pathway	0.000154882	Apelin Endothelial Signaling Pathway	8.7096×10^{-05}
Apoptosis Signaling	0.000151356	Apoptosis Signaling	0.00087096
Autophagy	5.62341×10^{-06}	Autophagy	0.00029512
CD40 Signaling	2.63027×10^{-05}	CD40 Signaling	4.0738×10^{-05}
Cell Cycle Checkpoints	7.07946×10^{-05}	Cell Cycle Checkpoints	2.138×10^{-05}
Colorectal Cancer Metastasis Signaling	0.000436516	Colorectal Cancer Metastasis Signaling	0.00077625
Death Receptor Signaling	9.12011×10^{-08}	Death Receptor Signaling	9.3325×10^{-05}
Deubiquitination	6.30957×10^{-12}	Deubiquitination	5.1286×10^{-05}
Endocannabinoid Developing Neuron Pathway	0.000301995	Endocannabinoid Developing Neuron Pathway	0.00075858
Gap Junction Signaling	0.000199526	Gap Junction Signaling	0.00057544
Glucocorticoid Receptor Signaling	0.000537032	Glucocorticoid Receptor Signaling	2.3988×10^{-05}
HGF Signaling	2.39883×10^{-07}	HGF Signaling	4.7863×10^{-05}
IL-10 Signaling	2.95121×10^{-05}	IL-10 Signaling	0.00079433
IL-3 Signaling	1.04713×10^{-05}	IL-3 Signaling	0.00079433
Interferon gamma signaling	7.24436×10^{-07}	Interferon gamma signaling	1.8197×10^{-06}
Mitotic Prometaphase	2.45471×10^{-05}	Mitotic Prometaphase	3.8905×10^{-05}
Molecular Mechanisms of Cancer	1.86209×10^{-06}	Molecular Mechanisms of Cancer	9.5499×10^{-06}
Natural Killer Cell Signaling	6.45654×10^{-05}	Natural Killer Cell Signaling	0.00045709
Neuroinflammation Signaling Pathway	0.000186209	Neuroinflammation Signaling Pathway	4.4668×10^{-05}
p53 Signaling	0.000147911	p53 Signaling	0.00058884
Paxillin Signaling	3.38844×10^{-05}	Paxillin Signaling	0.0002138
PI3K/AKT Signaling	2.75423×10^{-08}	PI3K/AKT Signaling	0.00050119
Protein Ubiquitination Pathway	1.94984×10^{-05}	Protein Ubiquitination Pathway	0.00026915
Pyroptosis Signaling Pathway	0.000549541	Pyroptosis Signaling Pathway	7.9433×10^{-05}
Regulation of TP53 Activity through Phosphorylation	0.00017378	Regulation of TP53 Activity through Phosphorylation	0.00041687
Renin-Angiotensin Signaling	0.000125893	Renin-Angiotensin Signaling	3.7154×10^{-06}
Ribonucleotide Reductase Signaling Pathway	1.51356×10^{-06}	Ribonucleotide Reductase Signaling Pathway	0.00011749
Role of NFAT in Cardiac Hypertrophy	0.000512861	Role of NFAT in Cardiac Hypertrophy	2.9512×10^{-05}
UVA-Induced MAPK Signaling	0.000398107	UVA-Induced MAPK Signaling	0.00058884

Table S4B. List of the microarray Canonical Pathways and their respective p-values. Sorted by p-value.

Microarray Ingenuity Canonical Pathways	p-value
Antigen Presentation Pathway	8.1283×10^{-06}
Role of Macrophages, Fibroblasts and Endothelial Cells in Rheumatoid Arthritis	2.5704×10^{-05}
Cardiac Hypertrophy Signaling (Enhanced)	5.2481×10^{-05}
Role of MAPK Signaling in the Pathogenesis of Influenza	0.00020417
P2Y Purinergic Receptor Signaling Pathway	0.00024547
Role of IL-17F in Allergic Inflammatory Airway Diseases	0.00026303
Crosstalk between Dendritic Cells and Natural Killer Cells	0.00035481
HMGB1 Signaling	0.00038905
UVC-Induced MAPK Signaling	0.00040738
Adrenomedullin signaling pathway	0.00047863
Mitotic G2-G2/M phases	0.00047863
Role of IL-17A in Arthritis	0.00075858
Regulation of TP53 Expression and Degradation	0.00085114
Mitotic G1 phase and G1/S transition	0.00095499
PI Metabolism	0.00097724
GADD45 Signaling	0.001
Retinoic acid Mediated Apoptosis Signaling	0.001

Table S4C. List of RNA-Seq Canonical Pathways and their respective p-value. Sorted by p-value.

RNA-Seq Ingenuity Canonical Pathways	p-value
Class I MHC mediated antigen processing and presentation	3.98107×10^{-18}
Protein Sorting Signaling Pathway	3.98107×10^{-15}
RHO GTPase cycle	3.98107×10^{-14}
HER-2 Signaling in Breast Cancer	1.25893×10^{-11}
Interferon alpha/beta signaling	1.99526×10^{-11}
ISG15 antiviral mechanism	1.99526×10^{-11}
Histone Modification Signaling Pathway	3.16228×10^{-11}
Processing of Capped Intron-Containing Pre-mRNA	4.36516×10^{-10}
Nuclear Cytoskeleton Signaling Pathway	2.04174×10^{-09}
p75 NTR receptor-mediated signaling	4.57088×10^{-09}
Senescence Pathway	6.76083×10^{-09}
ERK/MAPK Signaling	1.77828×10^{-08}
Intra-Golgi and retrograde Golgi-to-ER traffic	4.57088×10^{-08}
Th1 and Th2 Activation Pathway	8.91251×10^{-08}
COPII-mediated vesicle transport	1.07152×10^{-07}
MSP-RON Signaling in Cancer Cells Pathway	1.28825×10^{-07}
ISGylation Signaling Pathway	1.38038×10^{-07}
Interleukin-15 signaling	1.90546×10^{-07}

Signaling by CSF1 (M-CSF) in myeloid cells	$2.5704×10^{-07}$
Chronic Myeloid Leukemia Signaling	$3.54813×10^{-07}$
Signaling by NTRK1 (TRKA)	$3.63078×10^{-07}$
Interferon Signaling	$4.46684×10^{-07}$
Mitotic Prophase	$5.12861×10^{-07}$
SUMOylation of DNA replication proteins	$5.88844×10^{-07}$
Neddylation	$7.58578×10^{-07}$
DDX58/IFIH1-mediated induction of interferon-alpha/beta	$8.12831×10^{-07}$
Cytosolic sensors of pathogen-associated DNA	$8.51138×10^{-07}$
Coronavirus Pathogenesis Pathway	$9.33254×10^{-07}$
MicroRNA Biogenesis Signaling Pathway	$1.12202×10^{-06}$
PPARα/RXRα Activation	$1.28825×10^{-06}$
SUMOylation of DNA damage response and repair proteins	$1.38038×10^{-06}$
Th1 Pathway	$2.75423×10^{-06}$
Unfolded protein response	$3.38844×10^{-06}$
Interleukin-3, Interleukin-5 and GM-CSF signaling	$3.89045×10^{-06}$
Caveolar-mediated Endocytosis Signaling	$4.0738×10^{-06}$
Signaling by Erythropoietin	$4.57088×10^{-06}$
Mitotic Metaphase and Anaphase	$4.89779×10^{-06}$
Myelination Signaling Pathway	$5.01187×10^{-06}$
Formation of WDR5-containing histone-modifying complexes	$5.24807×10^{-06}$
Necroptosis Signaling Pathway	$5.37032×10^{-06}$
PDGF Signaling	$5.7544×10^{-06}$
FLT3 Signaling in Hematopoietic Progenitor Cells	$6.16595×10^{-06}$
JAK/STAT Signaling	$6.16595×10^{-06}$
OAS antiviral response	$6.16595×10^{-06}$
SUMOylation of ubiquitinylation proteins	$6.16595×10^{-06}$
RHO GTPases Activate Formins	$7.24436×10^{-06}$
IL-7 Signaling Pathway	$8.31764×10^{-06}$
Neuregulin Signaling	$9.12011×10^{-06}$
Prolactin Signaling	0.00001
Role of PKR in Interferon Induction and Antiviral Response	$1.1749×10^{-05}$
COPI-mediated anterograde transport	$1.31826×10^{-05}$
G alpha (12/13) signaling events	$1.31826×10^{-05}$
Neutrophil degranulation	$1.34896×10^{-05}$
RANK Signaling in Osteoclasts	$1.38038×10^{-05}$
Th2 Pathway	$1.38038×10^{-05}$
Signaling by VEGF	$1.41254×10^{-05}$
Prostate Cancer Signaling	$1.47911×10^{-05}$

GM-CSF Signaling	1.51356×10^{-05}
NGF Signaling	1.54882×10^{-05}
Thrombin Signaling	1.54882×10^{-05}
Pancreatic Adenocarcinoma Signaling	1.58489×10^{-05}
ID1 Signaling Pathway	1.90546×10^{-05}
EGF Signaling	2.13796×10^{-05}
SUMOylation of chromatin organization proteins	2.13796×10^{-05}
Type II Diabetes Mellitus Signaling	2.51189×10^{-05}
April Mediated Signaling	2.5704×10^{-05}
Endocannabinoid Cancer Inhibition Pathway	2.5704×10^{-05}
RAB GEFs exchange GTP for GDP on RABs	2.81838×10^{-05}
Signaling by NOTCH1	2.81838×10^{-05}
Virus Entry via Endocytic Pathways	3.01995×10^{-05}
Huntington's Disease Signaling	3.0903×10^{-05}
Endoplasmic Reticulum Stress Pathway	3.23594×10^{-05}
SUMOylation of SUMOylation proteins	3.80189×10^{-05}
Telomerase Signaling	4.0738×10^{-05}
Cellular response to heat stress	4.46684×10^{-05}
Melanocyte Development and Pigmentation Signaling	5.37032×10^{-05}
Pyridoxal 5'-phosphate Salvage Pathway	5.7544×10^{-05}
Pre-NOTCH Expression and Processing	5.88844×10^{-05}
GNRH Signaling	6.0256×10^{-05}
CLEAR Signaling Pathway	6.60693×10^{-05}
Translocation of SLC2A4 (GLUT4) to the plasma membrane	6.60693×10^{-05}
Mitotic Telophase/Cytokinesis	7.4131×10^{-05}
ATM Signaling	7.58578×10^{-05}
Opioid Signaling Pathway	7.94328×10^{-05}
NLR signaling pathways	8.12831×10^{-05}
Interleukin-2 family signaling	8.51138×10^{-05}
Role of RIG1-like Receptors in Antiviral Innate Immunity	8.51138×10^{-05}
Role of Tissue Factor in Cancer	8.51138×10^{-05}
SUMOylation of RNA binding proteins	8.51138×10^{-05}
TNFR1 Signaling	8.51138×10^{-05}
NF-κB Activation by Viruses	8.91251×10^{-05}
IL-2 Signaling	9.77237×10^{-05}
Estrogen Receptor Signaling	0.000102329
Immunogenic Cell Death Signaling Pathway	0.000102329
AMPK Signaling	0.000104713
IL-1 Signaling	0.000104713

Macrophage Classical Activation Signaling Pathway	0.000104713
TNF signaling	0.000104713
Signaling by ALK	0.000112202
Interleukin-9 signaling	0.00011749
LPS-stimulated MAPK Signaling	0.00011749
Protein Kinase A Signaling	0.00011749
Epithelial Adherens Junction Signaling	0.000120226
Glucose metabolism	0.000131826
Production of Nitric Oxide and Reactive Oxygen Species in Macrophages	0.000131826
Role of BRCA1 in DNA Damage Response	0.000131826
B Cell Activating Factor Signaling	0.000147911
Oncostatin M Signaling	0.000147911
Signaling by SCF-KIT	0.000147911
IL-6 Signaling	0.000165959
Angiopoietin Signaling	0.00018197
ERB2-ERBB3 Signaling	0.000190546
Transcriptional activity of SMAD2/SMAD3:SMAD4 heterotrimer	0.000190546
RAC Signaling	0.000213796
Docosahexaenoic Acid (DHA) Signaling	0.000229087
Cholecystokinin/Gastrin-mediated Signaling	0.000234423
Regulation of lipid metabolism by PPARalpha	0.000234423
MYC Mediated Apoptosis Signaling	0.000245471
PD-1, PD-L1 cancer immunotherapy pathway	0.000245471
Actin Cytoskeleton Signaling	0.000251189
PTEN Signaling	0.000251189
IL-9 Signaling	0.00025704
Eicosanoid Signaling	0.000281838
Small Cell Lung Cancer Signaling	0.000288403
Hepatic Fibrosis Signaling Pathway	0.000316228
Insulin Receptor Signaling	0.000316228
Insulin Secretion Signaling Pathway	0.000338844
IL-27 Signaling Pathway	0.000346737
Remodeling of Epithelial Adherens Junctions	0.000346737
RHO GTPases Activate WASPs and WAVEs	0.000346737
DNA Double-Strand Break Repair by Homologous Recombination	0.000354813
Pancreatic Secretion Signaling Pathway	0.000363078
ERK5 Signaling	0.000371535
Acetylcholine Receptor Signaling Pathway	0.000380189
Endothelin-1 Signaling	0.000380189

Role of Hypercytokinemia/hyperchemokinemia in the Pathogenesis of Influenza	0.000389045
iNOS Signaling	0.00042658
Ephrin Receptor Signaling	0.000436516
RUNX1 regulates megakaryocyte differentiation and platelet function	0.000446684
TNFR2 Signaling	0.000446684
Toll Like Receptor 3 (TLR3) Cascade	0.000446684
TWEAK Signaling	0.000457088
HIPPO signaling	0.000467735
NRF2-mediated Oxidative Stress Response	0.000467735
Synaptogenesis Signaling Pathway	0.000501187
fMLP Signaling in Neutrophils	0.000512861
Hypoxia Signaling in the Cardiovascular System	0.000537032
Macropinocytosis Signaling	0.000537032
Mitochondrial L-carnitine Shuttle Pathway	0.00057544
Acute Phase Response Signaling	0.00060256
Integrin Signaling	0.00060256
PPAR Signaling	0.00060256
Cardiac Hypertrophy Signaling	0.000616595
Growth Hormone Signaling	0.000616595
Oxytocin Signaling Pathway	0.000616595
Growth hormone receptor signaling	0.000707946
IL-12 Signaling and Production in Macrophages	0.00074131
Intrinsic Pathway for Apoptosis	0.000758578
Lymphotoxin β Receptor Signaling	0.000758578
Metabolism of non-coding RNA	0.000758578
Transcriptional regulation of white adipocyte differentiation	0.000758578
4-1BB Signaling in T Lymphocytes	0.000794328
Oncogene Induced Senescence	0.000794328
Signaling by NOTCH2	0.000794328
Sumoylation Pathway	0.000831764
FAT10 Cancer Signaling Pathway	0.000851138
Signaling by EGFR	0.000851138
Signaling by NOTCH3	0.000851138
Acute Myeloid Leukemia Signaling	0.000870964
G Beta Gamma Signaling	0.000891251
Interleukin-1 family signaling	0.000891251
Signaling by MET	0.000891251
Androgen Signaling	0.000933254
Interleukin-10 signaling	0.000933254

IL-8 Signaling	0.000954993
